# Supplementary figures and images for: Multidrug Resistance Like Protein 1 Activity in Malpighian Tubules Regulates Lipid Homeostasis in Drosophila
Source: Membranes (Basel). 2021 Jun 8;11(6):432. doi: 10.3390/membranes11060432 (PMC8229909; doi:10.3390/membranes11060432)

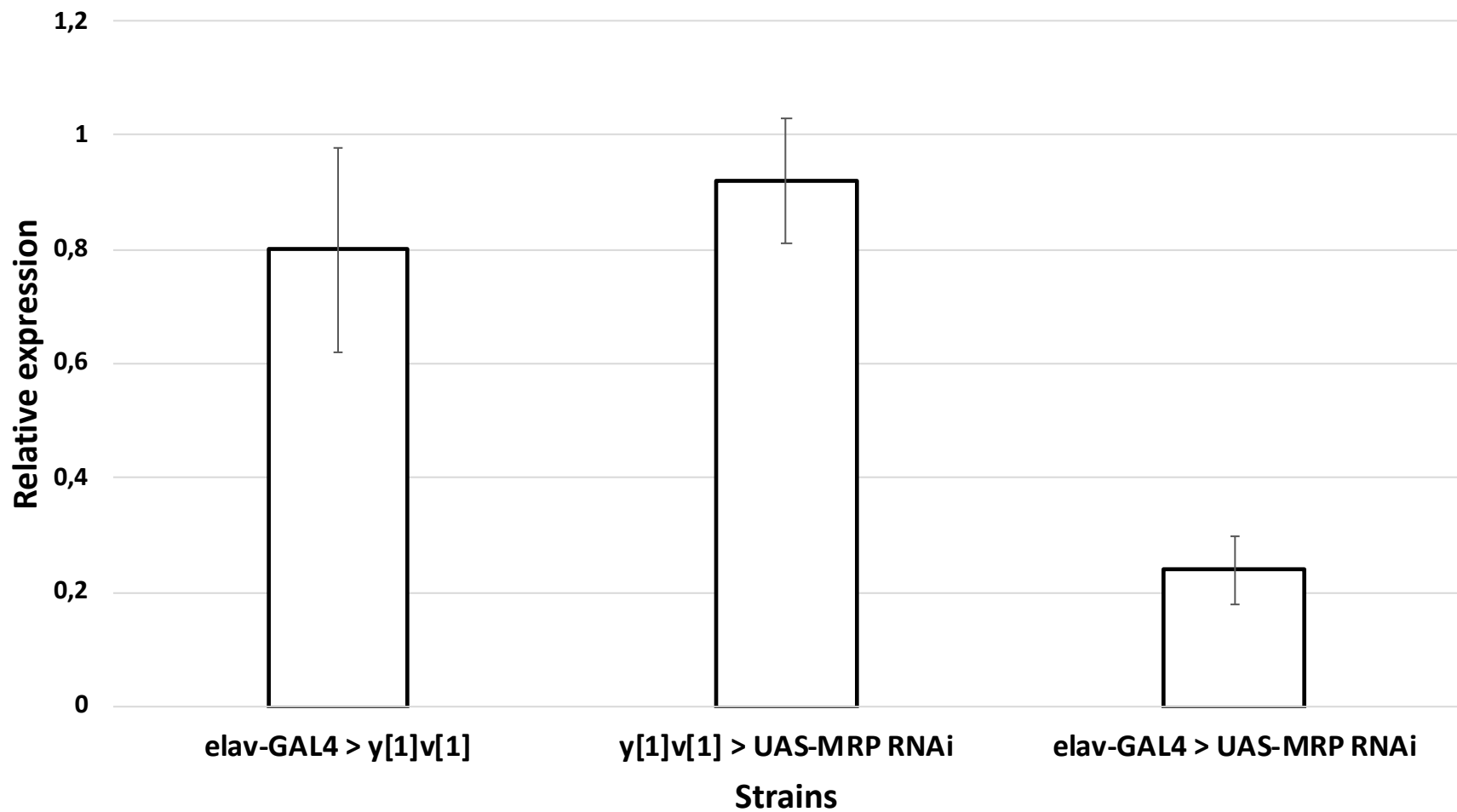

Supplement: Supplementary file 1 [file membranes-11-00432-s001.zip › membranes-1223938-supplementary.pdf]
